# Hand-held Colorimetry Sensor Platform for Determining Salivary α-Amylase Activity and Its Applications for Stress Assessment

**DOI:** 10.3390/s19071571

**Published:** 2019-04-01

**Authors:** Hsien-Yi Hsiao, Richie L. C. Chen, Chih-Chi Chou, Tzong-Jih Cheng

**Affiliations:** 1 Department of Bio-industrial Mechatronics Engineering, College of Bio-Resources and Agriculture, National Taiwan University, Taipei 100617, Taiwan; f92631001@ntu.edu.tw (H.-Y.H.); rlcchen@ntu.edu.tw (R.L.C.C.); R98631013@ntu.edu.tw (C.-C.C.); 2 Department of Biomedical Engineering, National Taiwan University Hospital, College of Medicine, National Taiwan University, Taipei 10002, Taiwan

**Keywords:** hand-held optical sensing meter, stress assessment, salivary α-amylase, colorimetry

## Abstract

This study develops a hand-held stress assessment meter with a chemically colorimetric strip for determining salivary α-amylase activity, using a 3,5 dinitrosalicylic acid (DNS) assay to quantify the reducing sugar released from soluble starch via α-amylase hydrolysis. The colorimetric reaction is produced by heating the strip with a mini polyester heater plate at boiling temperature to form a brick red colored product, which measured at 525 nm wavelength. This investigation describes in detail the design, construction, and performance evaluation of a hand-held α-amylase activity colorimeter with a light emitted diode (LED) and photo-detector with built-in filters. The dimensions and mass of the proposed prototype are only 120 × 60 × 60 mm^3^ and 200 g, respectively. This prototype has an excellent correlation coefficient (>0.995), comparable with a commercial ultraviolet–visible spectroscope, and has a measurable α-amylase activity range of 0.1–1.0 U mL^−1^. The hand-held device can measure the salivary α-amylase activity with only 5 μL of saliva within 12 min of testing. This sensor platform effectively demonstrates that the level of salivary α-amylase activity increases more significantly than serum cortisol, the other physiological stressor biomarker, under physiologically stressful exercise conditions. Thus, this work demonstrates that the hand-held α-amylase activity meter is an easy to use and cost-effective stress assessment tool for psychoneuroendocrinology research.

## 1. Introduction

Stress, stress-related diseases, and stress assessments are critical issues in clinical, psychological, biomedical, and sport medicine research. A stressor causes the two major biological stress systems, the Sympathetic–Adrenal–Medullary (SAM) system and the Hypothalamic–Pituitary–Adrenal system (HPA), to release hormones that control of most of the body’s internal organs, causing symptoms such as elevated heart and breathing rates [1]. Certain hormones in saliva, such as cortisol and catecholamines (norepinephrine, NE), that are released from the blood and are found in an unbound free state in saliva, are considered to be good stress biomarkers, and are widely monitored to measure endocrinological stress [2]. This is mainly because saliva sampling is non-invasive and not stressful, and does not require specialized personnel [1]. However, salivary catecholamines have concentrations several-fold lower than those of venous blood, and do not reflect the actuate changes in the blood catecholamines released by activation of SAM.

Several works have reported that salivary α-amylase (sAA) level is significantly positively correlated with plasma norepinephrine [1,2], and increases in response to both physical (e.g., exercise, heat and cold stress) and psychological (e.g., written examinations) stress stimuli via the SAM system, which is controlled and amplified with low levels of norepinephrine release in salivary glands [3]. The SAM system has a much faster response time of sAA secretion, from 1 min to a few minutes, than the endocrine system [4]. Therefore, sAA has been recommended to replace norepinephrine as a sensitive biomarker for stress via the SAM system, because it is very easy to monitor [5]. Alpha-amylase (EC 3.2.1.1) is a major protein component of saliva, and plays a key role in carbohydrate metabolism by hydrolyzing the 1,4-α-D-glycosidic linkages of starch components, glycogen, and various oligosaccharides to form maltose [6]. This major function of sAA is very important for food digestion. Therefore, the increase in sAA activity in response to stressful conditions, such as exercise, is considered to apply to intense energy expenditure.

Analytic approaches for assay of α-amylase activity fall into two main categories based on the carbohydrate hydrolysis mechanism. One group of methods measures the consumption of natural starch hydrolyzed by α-amylase. Some examples of this approach include viscometries by capillary viscometer [7], magnetoelastic cantilever [8] or piezoelectric transducer [9,10], electrochemically amperometry [11,12,13,14,15,16], potentiometry [17], colorimetry of starch-iodine [18,19] or a chromogenic substrate [20] color intensity, and liquid crystals-based sensing platform [21]. These methods generally have poor reproducibility, with large differences associated with distinct operators or slight protocol variations, because the attack speed of α-amylase is deeply influenced by varying molecular mass and branching degrees of heterogeneous starch macromolecules [22]. The other main category of methods overcomes those deficiencies by directly determining the amount of cleaved bonds in the substrate, the measure of which can be readily converted into International Units of enzymatic activity [23]. These methods are classified into two sub-categories, the chromogenic and saccharogenic approaches, according to analytical approach. In chromogenic methods, the release amounts of soluble dye are directly measured from covalently chemical-modified starch or maltosaccharide substrates, such as Cibacron Blue F3 G-A cross-linked starch [24] or nonreducing end-blocked 2-chloro-*p*-nitrophenyl maltohepotosides (Gal-G2-CNP) [25]. Chromogenic methods are simple, reliable, and sensitive for α-amylase determination, but are extremely expensive because they require a synthetic substrate and specific enzymes. Saccharogenic methods measure the release amount of reducing sugars from unmodified starch by colorimetric oxidant reagents, and include the Nelson–Somogyi assay [26] and 3,5-dinitrosalicyclic acid (DNS) assay [27]. These methods are reliable and low-cost, and are adopted extensively to measure α-amylase activity without any further modification in the procedure. However, they are not suitable for daily frequent use, because they require analytic instruments, and involve complicated analytic processes [27].

To improve the reproducibility, convenience, and cost in analytical applications, many portable optical-sensing instruments have been developed for process the monitoring and medical diagnosis fields, because they can be operated by lay people, and their results are obtained in the field and within a good time frame [28,29,30,31,32]. These hand-held colorimetric devices are generally miniaturized with light-emitting diodes (LEDs) as quasimonochromatic light sources for sensor-exciting and solid-state photodiodes for absorbance measurements. With appropriate bias and support circuits, the low-energy consumption, stability, and sensitivity of these optoelectronic devices have been shown to have the desired analytical performance, including accuracy.

This study develops a compact hand-held colorimetric device with a homemade disposable strip for determining sAA activity, and illustrates its feasibility for stress assessment. The sensing principle is based on the saccharogenic method, in which a DNS oxidant reagent is utilized to monitor the amount of reducing sugars released from soluble starch within the sAA hydrolysis at 37 °C. The hydrolytic solutions and reagents were injected into a homemade disposable strip, mixed with the vibration unit, and heated by a polyimide heater to form color at high temperature. The color results were then directly measured using an optical-sensing platform, to obtain the sAA activity. The experimental procedure addressed in detail the issues associated with the implementation of key components, disposable strip design, electronic and signal conditionings, and mechanism/optical chamber design. The analytical performance of the proposed device was compared with that of a commercial spectrophotometric instrument. Finally, the proposed hand-held device was then applied as a stress assessment platform to measure the sAA level in saliva collected under physiological exercise stress conditions and daily routine conditions.

## 2. Materials and Methods

### 2.1. Electronic Instruments and Machines for Developing a Hand-Hold Colorimeter

A power supply (GW instek, GPC-3030DQ, New Taipei City, Taiwan), oscilloscope (Tektronix, TDS2002, Taipei, Taiwan), function generator (GW instek, GFG-8015G, New Taipei City, Taiwan), multimeter (DHA, DMM-93B, Taipei, Taiwan), and frequency counter (Hewlett-Packard, 53131A, Palo Alto, CA, USA) were employed as electronic instruments to develop and verify the electronic hardware of the prototype. An in-circuit emulator for an 80S51 microprocessor (Ocean Sky Technology, TE-S51, New Taipei City, Taiwan) was used as the development platform. An UV/Vis spectroscope (JASCO, F-530, Tokyo, Japan) was adopted as a reference instrument to evaluate the consistency of the developed prototype. A laser cutting machine (LLC EZLaser, LCR-200R, Hsinchu, Taiwan) was utilized in the design and construction of the reactor chips by cutting polycarbonate from compact discs.

### 2.2. Chemicals and Reagents

Sodium hydroxide, potassium sodium tartrate, disodium hydrogenphosphate, and potassium dihydrogenphosphate were obtained from Nacalai Tesque, Kyoto, Japan. The 3,5-dinitrosalicylic acid was acquired from Sigma-Aldrich, St. Louis, MO, USA. α-amylase (EC 3.2.1.1, 20 U mg^−1^, from *Bacillus subtilis*) and soluble starch (solubility 20 mg mL^−1^ at 25 °C, pH 4.0–6.0, from potato) were purchased from Wako, Osaka, Japan. All chemicals were of analytical-reagent grade, and were used as received

Starch stock solution (10 mg mL^−1^) and α-amylase stock solution (1 mg mL^−1^) were prepared by dissolving starch and α-Amylase powders in 10 mM phosphate buffer (pH 6.90), respectively, and stored at 4 °C. The DNS reagent was prepared by dissolving 0.250 g 3,5-dinitrosalicylic acid and 75.0 g potassium sodium tartrate in 50 mL, 2 M NaOH solution, and diluted with de-ionized (DI) water to total volume 250 mL. Buffers and solutions were prepared with deionized water with conductivity < 1 μS cm^−1^.

### 2.3. Measurement of α-Amylase Activity

Amylase activity was determined by measuring the amount of reducing sugars released from soluble starch using alkaline DNS oxidant reagent [33]. Thirty microliters of 10 mg mL^−1^ soluble starch solution was mixed with 20 μL of α-amylase solution or salivary sample, and incubated for 1 min at 37 °C. The hydrolyzing solution was injected into a home-made disposable strip, and the reaction was stopped with the addition of 150 μL DNS reagent. The mixture was mixed uniformly with the micro-eccentric vibrator, and heated with flexible polyester heater (above 80 °C) for 4 min to form color. The reducing groups were quantified at 525 nm with an optical-sensing platform for measuring α-amylase activity. Measurements were also performed on a blank without substrate but with α-amylase, and a control containing no α-amylase but with substrate, at the same time as the reaction mixtures. Each measurement procedure was performed several times, with measurements taken in duplicate.

### 2.4. Determination of Stress Levels

The salivary α-amylase activity and serum cortisol concentration were measured to assess the stress level of a volleyball player following exercises of different intensity. The male was 25 years old, and had no oral diseases. The aim of the experiment was explained to the subject, and consent was obtained after confirmation that he fully understood the experiment. Table 1 shows the self-training exercise conditions and the collection times of the blood samples. At conditions III and V, the blood was sampled 1 h after termination of the exercise. The subject voluntarily provided serum cortisol data from clinical reports of laboratory chemistry tests. The experimental procedure is briefly described as follows. All blood samples were accumulated in sterile tubes containing ethylenediaminetetraacetic acid (EDTA) and heparin. The total serum cortisol level of blood samples were measured in enzymatic mode with a clinical chemistry system (SIEMENS, Dimension RXL, Erlangen, Germany) in the UNION clinical laboratory, Taiwan.

The saliva was collected 30 min before blood sampling, immediately after blood sampling, and at 30-min intervals thereafter. To minimize error variance and chances of saliva secretion, food or drink intake must be avoided at least 1 h prior to sampling. Before sampling, the participant should clean his mouth with warm water, at least 10 min before collection, to eliminate any residues. The whole saliva samples were gathered using the “passive drool” method [34], and diluted with 20-fold volume of 10 mM phosphate buffer (pH 6.90). The α-amylase activity of the diluted saliva sample was determined with the hand-held colorimeter based on DNS coloration.

## 3. Results and Discussions

### 3.1. System Realization

**Figure 1:**
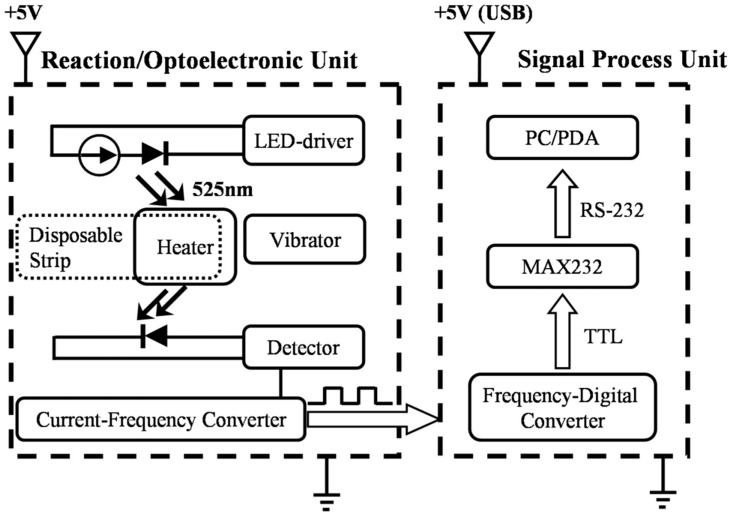
Function block diagram of hand-held photometer. PC: personal computer; PDA: personal digital assistant; MAX323: precision analog switches, MAXIM; TTL: transistor-transistor logic; LED: light-emitting diode.

The major objective of this prototype was to develop a low-cost portable colorimeter with a heat-resistant disposable strip, in order to obtain an easy-to-use piece of equipment intended for lay-personnel in analytical chemistry technology. Figure 1 illustrates a block diagram of the overall instrument design with a circuit. The two main components of the hand-held instrument are the reaction/optoelectronic and the electronic signal-processing units. The schematic diagram also describes the transformation of the signal from a light signal to a frequency-modulated signal and then to digital information feeding into the microprocessor (PC/PDA). Our previous work [35] revealed that the combination of a photo-detector with frequency-modulated digital output and a frequency-digital converter are cost-effective and easy to implement. The following sub-section describes the design and fabrication of disposable strips, as well as the heat/spatial configuration to support the light pathway. The later paragraphs describe in detail the two principal components of the device, and elucidate all aspects of their operation.

### 3.2. Manufacture of Disposable Strips

**Figure 2:**
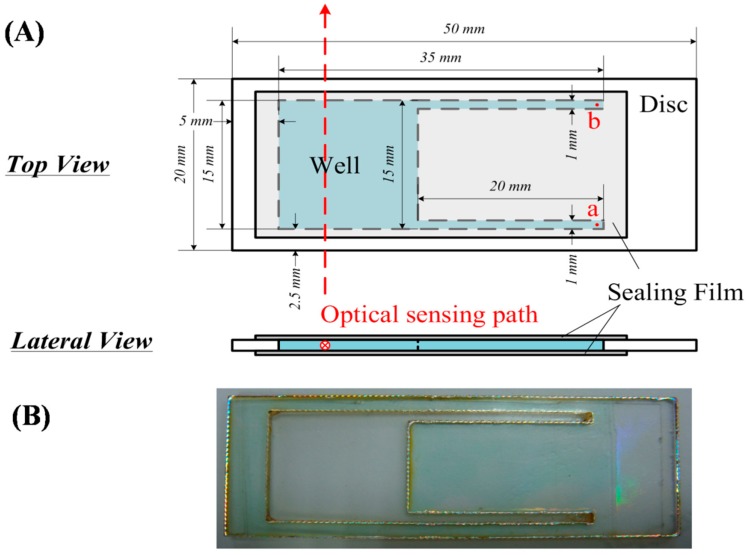
The schematic diagram (**A**) and outward view (**B**) of homemade disposable strip. The pinholes **a** and **b** are the reagent injection side and exhaust side, respectively.

To achieve the high temperature and alkaline conditions required for the DNS coloration reaction, the disposable strip was supported by a polycarbonate (PC) plate, because this has good chemical/heat resistance and visible-light transmittance. The polycarbonate plate was cut from a compact disc (thickness 1.20 mm) with a laser cutting machine (cutting rate: 1500 mm min^−1^; power: 40 W) into suitable schematic diagrams, shown in detail in Figure 2. The protective/reflective layer was then removed by tape. The upper and lower sides of transparent specimen were sealed with heat-resistant sealing film (UC-500, Axygen Bioscience, Inc., Union, CA, USA) to fabricate the homemade disposable strip, and the upper sealing film was punched with a syringe needle to form two pinholes at the terminal end of narrow channels to form the solution injection and gas exhaust sites. The spatial configuration of narrow channels and pinholes efficiently reduced the measurement error by removing from the solution the small bubbles that were produced from the DNS coloration at high temperatures or mixing procedures. To remove the bubbles, small vibrations were generated to push them into narrow channels and exhaust them via pinholes. Therefore, the absorbance of mixtures was monitored in real time with the optoelectronic platform through the cut surface of the disposable strip. The red dashed line in Figure 2A depicts the optical sensing path. The sealed structural design of this disposable strip improved the measurement repeatability by lowering the sample evaporation. The disposable strip had a transparency of about 20% (CV < 10%, n = 8), and required a total reagent volume of only 200 μL to decrease the sample usage and heating time (4 min, from 20 °C to 80 °C).

### 3.3. Reaction/Optoelectronic Hardware

**Figure 3:**
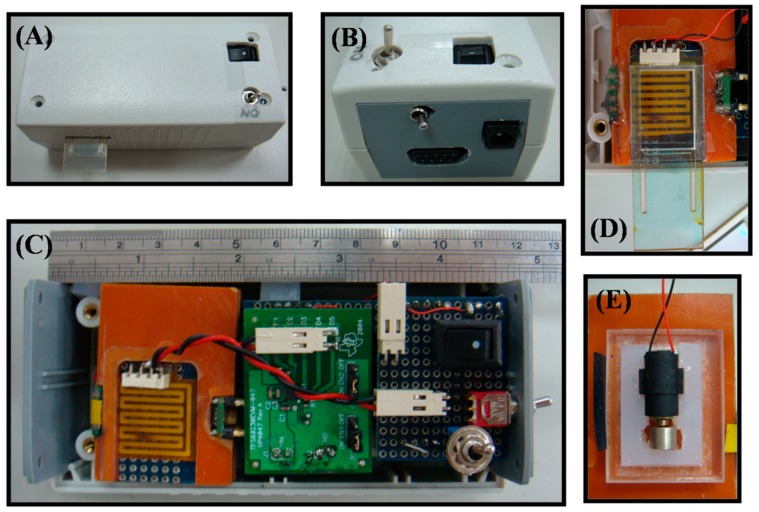
The prototype of hand-held photometer. (**A**,**B**) Outward of hand-held photometer and its side view, respectively. (**C**,**D**) The inside view of the hand-held photometer and a zoom-in view of disposable strip with polyester heater, respectively. (**E**) The zoom-in view of vibrator which adhesive on the back of bakelite plate.

Figure 3 shows a photograph of the established prototype and its sub-units. This hand-held colorimeter is assembled into a small plastic case (120 mm length, 60 mm width, and 60 mm height) to prevent ambient light from entering. The total net mass is approximately 200 g. To ensure thermal regulation and real-time detection for DNS coloration, the reaction/detection chamber(s) are fabricated from Bakelite (phenol formaldehyde resins), which has good heat resistance, optical insulation, and mechanical performance, using lathe processes. This chamber design ensures that the LED, sample cell, polyester heater, motor-based mini-vibrator, and photo-detector are placed close to each other, as illustrated in Figure 3C–E, thus exempting lenses and mirrors from optical conditioning requirement. The disposable strip is filled with the DNS reagents and samples, and then guided into reaction/detection chamber through the slot design. The reaction solution is mixed uniformly with the mini-vibrator located on the back of the Bakelite plate (Figure 3E), and heated by a flexible polyester heater to form the DNS coloration. This polyester-based heater with low power, higher watt density, and distributed wattage, closely located under the disposable strip, generates a uniform heat output to reduce dissipated heat. The absorbance of the mixture is measured from transmission at 525 nm with the LED and the photo-detector.

The light source of the colorimeter is an LED with a peak wavelength of 525 nm and a spectral line half-width of 30 nm (AM2520ZGC09, Kingbright Elec. Co., Ltd., New Taipei City, Taiwan). A 20 mA pulsed-current LED driver IC (TPS60230, Texas Instruments, TX, USA) is adopted to minimize the peak wavelength shift resulting from thermal dissipation from the LED, and thereby prevent fluctuation (short-term variation) and shift (long-term change) in light intensity. The transmission light is detected with a color/light sensor (TCS230, Texas Advanced Optoelectronic Solutions Inc., TX, USA). By combining silicon photodiodes and a current-to-frequency converter, the TCS230 sensor outputs a frequency-modulated signal (50% duty cycle) with a frequency value proportional to the intensity of light (irradiance). This frequency-modulated digital signal can be combined with a universal frequency-to-digital converter IC (UFDC-1M-16, International Frequency Sensor Association, Barcelona, Spain) to make a quasi-digital sensor that can interface with conventional digital circuits. The four types (R., G., B., and clear) of built-in filter in the TCS230 are responsible for the sensing selectivity of the wavelength windows. Owing to the transmission wavelength of the reagents (525 nm), the filter mode was set to green to minimize background interference. The selection of optical sensor is critical in developing the compact instrument due to considerations of hardware deployment and signal processes of both optical and electronic signals. The proposed device provides a simple and convenient solution to the system requirements. These optical-sensing elements are tightly embedded into the vertical surface of thermal insulation Bakelite chamber to lower the thermal interference from heated samples, and the driving and measuring circuits are separated from reaction/detection chamber to reduce thermal noise in the heating process.

### 3.4. Electronics, Signal Conditioning and Data Processing

As mentioned above, a UFDC was used as a simple converting interface between an optical sensor and a digital data processor. The communication protocol of the UFDC was set according to the Universal Asynchronous Receiver Transmitter (UART) convention by RS-232 protocol in binary-coded decimal (BCD) American standard code for information interchange (ASCII) format, and the data transfer rate (baud-rate) was set to 2400 bps. The UFDC outputs digital signals in transistor-transistor logic (TTL) evel (0/5 V) to communicate with a PC via a MAX232 IC (HIN 232CPZ, Intersil Americas Inc., Milpitas, CA, USA), which was adopted as a level converter from TTL to RS-232 level (±12 V). As well as simplifying the signal conditioning, UFDC improves the measurement performance, in terms of the detection range and the signal-to-noise ratio (SNR), beyond that of typical analog-to-digital (A/D) conversions in low-speed applications. The dynamic range of UFDC is not limited by the supply voltage of the converter integrated circuit (IC), and the signal is virtually immune to noise because it is in the frequency domain. All of the ICs, the optical components and polyester heater in this prototype were powered by two 5 V supplies. A future version of this colorimeter will thus be integrated into a single battery or USB-powered system.

A program in the LabView software (LabView 8.6.1, National Instruments, Austin, TX, USA) was developed to acquire data at a sampling rate of 30 Hz via an RS-232 virtual protocol in a USB hardware port of a PC; the data can then be processed on the same operating platform. The program plots the real-time measurements on a chart. The accuracy and precision of the hand-held instrument in measuring frequency values, displayed on a PC, were evaluated and confirmed with a universal counter (HP-53131A, Hewlett-Packard, Palo Alto, CA, USA).

### 3.5. Feasibility Verification of Hand-held Colorimeter for Determining α-amylase Activity

In biochemistry and analytical chemistry, samples are routinely cooled to improve measurement repeatability by reducing the solution evaporation or thermal interference from electronic elements. However, this cooling process requires additional cooling equipment, and is time-consuming and unsuitable for real-time monitors. To improve the measurement repeatability to monitor heated samples, this hand-held colorimeter incorporates an advanced mechanism with four main structural subunits: (1) Sealed disposable strip with pinholes to lower the sample evaporation; (2) Bakelite insulation material between heating source and electronic elements to reduce the thermal noise effects; (3) closely integrated disposable strip and heater to reduce the power dissipation; and (4) a mini-vibrator adhesive on the back of the bakelite plate to provide movement energy for small bubbles to dissipate via the pinholes in the heating process. The optical signal shift and variation coefficient of the heated samples (DNS solution) are only 0.32 Hz min^−1^ and 0.20%, respectively, after the hand-held colorimeter has warmed up for about 20 s. On the basis of these values, the hand-held colorimeter is suitable for use as a real-time monitor for heated samples.

**Figure 4:**
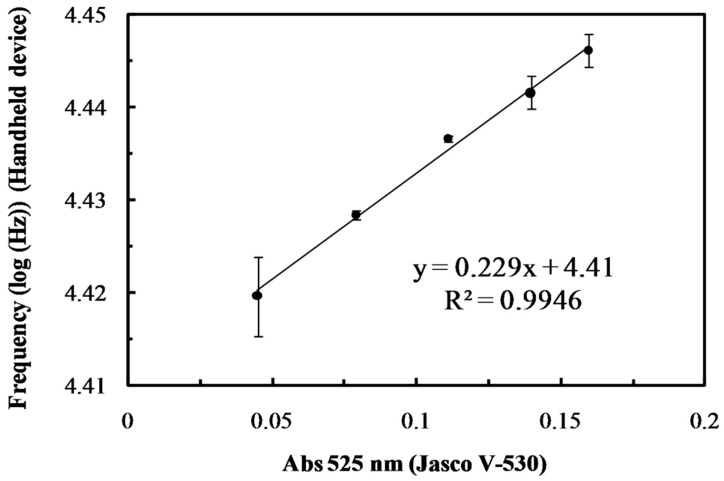
Correlation between the proposed hand-held photometer (vertical axis) and a commercial laboratory UV/Vis spectroscopy (horizontal axis) for 0.1–1.0 U mL^−1^ of α-amylase. Twenty microliters of α-amylase solution and 30 mL of 10 mg mL^−1^ starch solution were mixed and incubated for 1 min at 37 °C. The mixtures and 150 L DNS reagent were injected into the homemade disposable strip and heated above 80 °C for 4 min with a polyester heater.

To shorten the time taken to heat from room temperature to reaction temperature (above 80 °C), the disposable strips are designed with a flat structure to raise the heating rate by increasing the heating surface and reducing the sample filled volume. Additionally, a low-power flexible polyester heater is utilized to increase the density and uniformity of the heating output. The heating time of the 200 μL sample in the strip from 25 °C to 80 °C and 80 °C to 94 °C were 4 min and 3 min, respectively, and then the temperature was maintained at 94 °C with heat dissipation to prevent sample boiling. The DNS coloration for reducing sugars was fully formed above 80 °C within 4 min, and was monitored at 525 nm. The developed hand-held photometer yields results that correlate (R^2^ > 0.994) closely with those obtained using a commercialized ultraviolet–visible spectrometer, with a highly sensitive but expensive photomultiplier tube (PMT), photo-detector, from samples diluted 10-fold with DI water, as shown in Figure 4. The experimental results reveal that the developed hand-held instrument can feasibly be applied in quantitative assay of photosensing (bio)chemistry.

**Figure 5:**
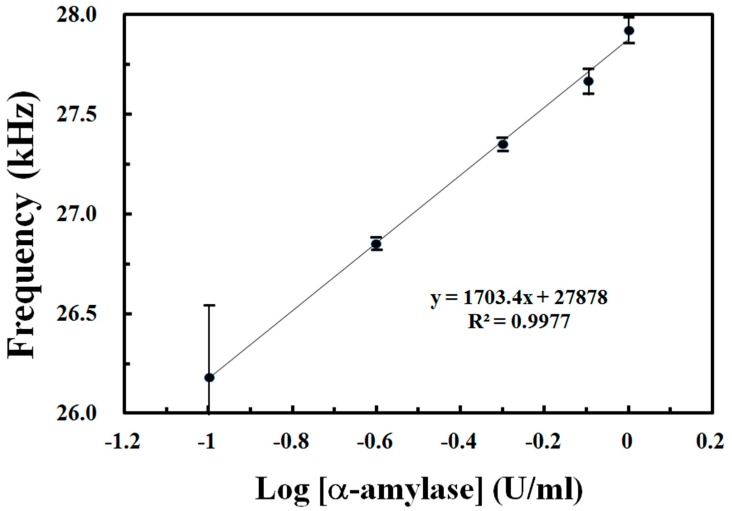
Calibration curve of 0.1–1.0 U mL^−1^ α-amylase hydrolyzed starch solution with 3,5 dinitrosalicylic acid (DNS) reagent. Other detailed operational conditions are shown in Figure 4.

Figure 5 plots the frequency output of the photodetector for the absorbance response of DNS reagents to various concentrations of α-amylase. The linear detection range of the hand-held photometer for α-amylase activity is 0.1–1.0 U mL^−1^, and the response reaches saturation when the α-amylase activity reaches 2.0 U mL^−1^ (not shown here). The calibration curve is linear to α-amylase activity because it is located in the low activity range. The achieved detection limit and sensitivity values were 0.1 U mL^−1^ and 1703.4 Hz (log U mL^−1^)^−1^, respectively. The resolution of the proposed system with DNS reagent for α-amylase is as low as 5.9 × 10^−4^ log U ml^−1^, given a 1 Hz precision of the instrument adopted with the UFDC module. As shown in Table 1, the proposed α-amylase meter performs with an excellent sensitivity and good sample volume requirement, as well as a slightly better detection range than an iodine-based colorimetry [19]. Despite the meter’s detection range being lower than some electrochemical methods [11,16,17], its excellent sensitivity will overcome this weakness when it used in higher concentration conditions by dilution processes. The linear detection range of this prototype for α-amylase activity was below the diurnal range of sAA activity (10–300 U ml^−1^), but is still usable to quantitatively determine sAA levels in a sample to within the predetermined linear α-amylase activity range (0.1–1 U ml^−1^). 

### 3.6. Application to Stress Evaluation

Stress can be measured using several methods, including psychological tests, measurements of hormonal and cardiovascular responses, and other physiological parameters. Compared with measurements of physiological parameters such as heart rate and blood pressure, the monitors of stress-related indicators such as cortisol and norepinephrine are easy to quantify, and show significant differences in response to eustress and distress [36]. This investigation selected serum cortisol (reference method) and sAA as stress-related biomarkers of HPA and SAM activity in response to daily lifestyle and stressful exercise. The experimental results demonstrate that the proposed sAA activity sensor platform performs better than serum cortisol for distinguishing stress levels.

Table 2 lists the blood sample collection times, exercise training conditions, and serum cortisol levels. The serum cortisol level was higher in the morning (Exp II) than in the afternoon (Exp IV and VI) for the normal daily routine, because cortisol is a steroid hormone that is secreted diurnally [37] in response to pulsatile trophic hormone stimulation; cortisol levels peak early prior to awakening, and decreasing progressively during the day to reach low levels in the evening. This appearance is also confirmed by the comparison between the morning conditions (Exp I and II) and afternoon conditions (Exp III ~ VI), despite the different intensity of exercises shown in Table 2. The serum cortisol levels sampled 1 h after exercise (Exp III, V) were significantly higher than those in the normal daily routine (Exp IV, VI) at similar collection times (13:40), but the serum cortisol level collected in the morning with over 8 hr of rest after exercise (Exp I) was similar to that in the normal daily routine (Exp II). This finding indicates that the serum cortisol after rest was metabolized and self-regulated via the negative feedback of HPA axis whereby elevated circulating cortisol levels lead to diurnal concentration [37], and the serum cortisol level does not fully reflect the fatigue feeling of prolonged exercise. Therefore, the serum cortisol level is not a good reference indicator to consider the psychological or physiological stress of subjects. Consequently, this study seeks to demonstrate that the proposed sAA meter could be a valuable tool to assess fatigue levels. 

**Figure 6:**
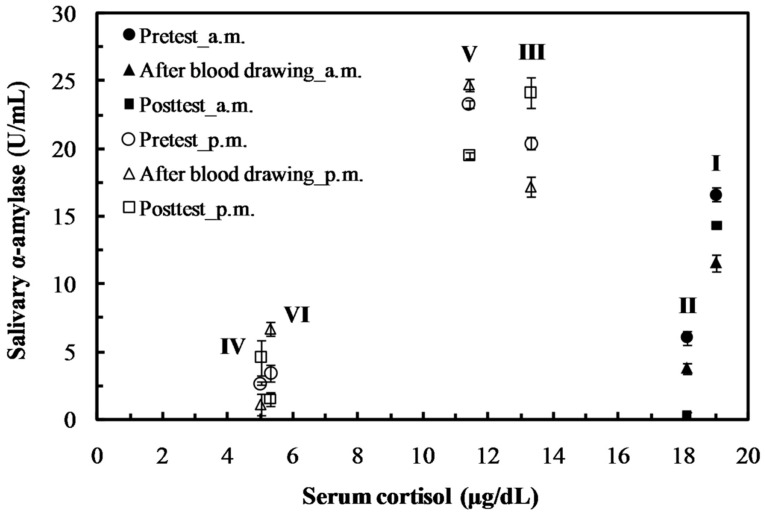
Serum cortisol concentration (horizontal axis) and salivary α-amylase (vertical axis) response to physiological stressful exercise conditions (Exp I, III, and V) and daily routine conditions (Exp II, IV, and VI). The solid circles (Exp I, II) and open circles (Exp III–VI) are blood samples collected in the morning and afternoon, respectively.

Unlike serum cortisol, the non-hormonal sAA activities sampled from the normal daily routine conditions (Exp. II, IV, and VI in Figure 6) were similar (<8 U mL^−1^) at different collection times (morning or afternoon) [5,38], and its activity could be a good reference indicator for assessing psychological or physiological stress of subjects. To evaluate the psychological stress of subjects during the blood collection procedure, the saliva was gathered 30 min before blood sampling, immediately after blood sampling, and at intervals of 30 min thereafter. The sAA results from these samples were filled in at the same intervals (Figure 6), indicating that the blood collection procedure did not significantly influence the psychological stress of subjects. The sAA activity of salivary samples collected in one hour of low- and high-intensity tests (Exp. III and V, respectively) after stressful prolonged exercise were three times those under normal daily routine (Exp II, IV, and VI), even if the subject took an overnight rest after a super-loading exercise (Exp I). The sAA activity determined by the proposed system can effectively and quantitatively distinguish physiological stress levels, and avoid the diurnal effect found in serum cortisol.

As indicated in Exp. I and II of Figure 6, two subjects who underwent significantly different loading exercises had significantly sAA levels, three-fold different as measured by the proposed meter, but had almost equal serum cortisol levels (value difference < 5%). The recovery time of sAA response for physiological exercise was much longer than the tens of minutes found for psychological stressful conditions, i.e., the Trier Social Stress Test [5,39]. This phenomenon indicates that the sAA secretion is released from the salivary glands by controlling the stimulation of plasma norepinephrine, which is a catecholamine with multiple roles, including neurotransmitter and hormone. In response to physiological and psychological stressors, this neurotransmitter is immediately released from the sympathetic neurons affecting the salivary glands to secrete sAA (about a few minutes) and recovers to normal concentration (about tens of minutes) by signal termination of norepinephrine reuptake. However, the performance of acute norepinephrine reuptake inhibition may decrease in a high ambient temperature whilst at rest and during exercise [40]. The exercise-induced increase of norepinephrine is delayed, or the onset of central fatigue is accelerated, to control body temperature during prolonged exercise (1–3 h) [41,42]. Therefore, the sAA is not only a sensitive biomarker for evaluating physiological and psychological stress, but can also be utilized to evaluate central fatigue after prolonged exercise [43]. This result implies that the proposed sAA meter might be directly useful for evaluating the physical resilience (capacity of physiological recovery) or accumulation effect of fatigue.

## 4. Conclusions

In summary, this study develops a compact analytic system for α-amylase activity based on colorimetry, and shows its feasibility in stress assessment with salvia. The linear detection range of α-amylase activity is 0.1–1 U mL^−1^; it also shows good correlation with commercial ultraviolet–visible spectroscopy. The developed prototype has satisfactory instrumentation performance, and is therefore very attractive as a device that exploits photometry to determine quantities of α-amylase activity in the field. The hand-held device has a small sample consumption (about 5 μL), low cost, and convenient measurement, and is applied to physiological exercise stress assessment by measuring the α-amylase level in the whole saliva. The sAA activity determined by the proposed system can effectively and quantitatively distinguish physiological stress levels, but does not exhibit the diurnal effect found in serum cortisol. The sAA activity of saliva in stressful high-intensity exercise was thrice that under the normal daily routine, even if the subject took an overnight rest. These results demonstrate that the proposed sAA meter considered is a sensitive biomarker meter not only for short-term stressful stimuli, but also for long-term fatigue following prolonged exercise. This convenient hand-held device will be a valuable tool in the evaluation of stress and central fatigue in the future.

## Figures and Tables

**Table 1:** Comparison between the analytical performances obtained in the current study and other methods reported in literature. QCM-D: quartz crystal microbalance with dissipation monitoring; BIA-AD: batch injection analysis system with amperometric detection; C: α-amylase activity (U mL^−1^).

Reference [method]	Detection Limit (U mL^−1^)	Detection Range (U mL^−1^)	Sensitivity (Response / U mL^−1^)	Reaction Time (min.)	Sample Volume (mL)
Yamaguchi et al., 2003 [amperometry]	-	0~30	-	-	-
Yamaguchi et al., 2004 & 2006* [colorimetry]	- -	0~200 10~140*	- -	2.5 -	5 30*
Wu et al., 2007 [magnetoelastic]	-	75~125	-	-	-
Sasaki et al., 2008 [QCM-D]	-	0.001~1	0.88 log C	-	-
Mahosenaho et al., 2010 [amperometry]	5	5~250	0.997 C	-	-
Zhang et al., 2014 [chronocoulometry]	0.022	0.03~3	5.20 C	-	-
Zhang et al., 2015 [potentiometry]	0.12	30~1000	0.12 C	5	25
Wang et al., 2015 [amperometry]	0.02	0~1	0.01276 C	15	-
Dutta et al., 2016 [colorimetry, paper-based]	-	0.01~0.11	0.003 C	-	-
Garcia et al., 2018 [amperometry, BIA-AD]	0.05	0.5~10	48.8 C	0.5	15
Garcia et al., 2018 [amperometry]	1.1	100~1200	10.7 log C	20	15
Ma et al., 2019 [liquid crystal]	0.015	-	-	-	-
This work [colorimetry]	0.1	0.1~1.0	1703.4 log C	12	5

**Table 2:** The collection conditions and serum cortisol levels of six blood samples.

Number	Collection Time	Collection Status	Serum Cortisol (μg/dL)
I	09:25	More than five hours of continuous sport last night	19.0
II	09:50	Normal daily routine	18.1
III	13:40	1 hour of low intensity race at noon	13.3
IV	13:30	Normal daily routine	5.0
V	13:40	1 hour of high intensity race at noon	11.4
VI	12:45	Normal daily routine	5.3
